# The Association of a classical left bundle Branch Block Contraction Pattern by vendor-independent strain echocardiography and outcome after cardiac resynchronization therapy

**DOI:** 10.1186/s12947-019-0160-4

**Published:** 2019-05-21

**Authors:** Kasper Emerek, Daniel J. Friedman, Peter L. Sørensen, Steen M. Hansen, Jacob M. Larsen, Niels Risum, Anna Margrethe Thøgersen, Claus Graff, Brett D. Atwater, Joseph Kisslo, Peter Søgaard

**Affiliations:** 1 Department of Medicine, Division of Cardiology, Duke University Hospital, Durham, NC USA; 2 Department of Clinical Medicine, Aalborg University, Aalborg, Denmark; 3 Department of Health Science and Technology, Aalborg University, Aalborg, Denmark; 4 Unit of Epidemiology and Biostatistics, Aalborg University Hospital, Aalborg, Denmark; 5 Department of Cardiology, Aalborg University Hospital, Aalborg, Denmark; 6 Department of Cardiology, Rigshospitalet, Copenhagen, Denmark

**Keywords:** Heart failure, Left bundle branch block, Cardiac resynchronization therapy, Speckle-tracking echocardiography

## Abstract

**Background:**

The association of a Classical left bundle branch block (LBBB) contraction pattern and better outcome after cardiac resynchronization therapy (CRT) has only been studied using vendor-specific software for echocardiographic speckle-tracked longitudinal strain analysis. The purpose of this study was to assess whether a Classical LBBB contraction pattern on longitudinal strain analysis using vendor-independent software is associated with clinical outcome in CRT recipients with LBBB.

**Methods:**

This was a retrospective cohort study including CRT recipients with LBBB, heart failure, and left ventricular (LV) ejection fraction ≤35%. Speckle-tracked echocardiographic longitudinal strain analysis was performed retrospectively on echocardiograms using vendor-independent software. The presence of a Classical LBBB contraction pattern was determined by consensus of two readers. The primary end point was a composite of time to death, heart transplantation or LV assist device implantation. Secondary outcome was ≥15% reduction in LV end-systolic volume. Intra- and inter-reader agreement of the longitudinal strain contraction pattern was assessed by calculating Cohen’s κ.

**Results:**

Of 283 included patients, 113 (40%) were women, mean age was 66 ± 11 years, and 136 (48%) had ischemic heart disease. A Classical LBBB contraction pattern was present in 196 (69%). The unadjusted hazard ratio for reaching the primary end point was 1.93 (95% confidence interval, 1.36–2.76, *p* < 0.001) when comparing patients without to patients with a Classical LBBB contraction pattern. Adjusted for ischemic heart disease and QRS duration < 150 milliseconds the hazard ratio was 1.65 (95% confidence interval, 1.12–2.43, *p* = 0.01). Of the 123 (43%) patients with a follow-up echocardiogram, 64 of 85 (75%) of patients with a Classical LBBB contraction pattern compared to 13 of 38 (34%) without, had ≥15% reduction in LV end-systolic volume (*p* < 0.001). Cohen’s κ were 0.86 (95% confidence interval, 0.71–1.00) and 0.42 (95% confidence interval, 0.30–0.54) for intra- and inter-reader agreement, respectively.

**Conclusion:**

Using vendor-independent strain software, a Classical LBBB contraction pattern is associated with better outcome in CRT recipients with LBBB, but inter-reader agreement for the classification of contraction pattern is only moderate.

## Introduction

Cardiac resynchronization therapy (CRT) is an effective treatment for patients with heart failure with reduced ejection fraction and left bundle branch block (LBBB). [1, 2] Unfortunately, a substantial proportion of patients receiving CRT do not improve their functional or echocardiographic status. [3, 4] In recent years, a Classical (also called Typical) LBBB contraction pattern on two-dimensional speckle-tracked echocardiographic longitudinal strain analysis has been associated with improved echocardiographic function and survival free of heart transplantation or left ventricular (LV) assist device implantation in CRT recipients independent of QRS duration and ischemic etiology. [5, 6] However, these studies were performed using vendor-specific software for analysis of prospectively acquired high frequency speckle-tracked strain images, and the results have not been replicated using vendor-independent software analysis of images acquired using standard acquisition protocols. While vendor-independent software correlates well with different vendor-specific software for assessment of global longitudinal strain, [7, 8] assessment of specific contraction patterns may not be equally feasible. The use of vendor-independent software can be required in certain situations, e.g. in health centers with multiple vendors of echocardiography systems, or if retrospective analysis on images saved in Digital Imaging and Communications in Medicine (DICOM) format is needed.

The aim of this study was to assess whether the presence of a Classical LBBB contraction pattern on speckle-tracked longitudinal strain analysis using vendor-independent strain software was associated with better survival and echocardiographic response in CRT recipients with LBBB.

## Methods

This was a retrospective cohort study performed at Duke University Hospital, a high-volume tertiary medical center.

### Study population

All patients who received a CRT with defibrillator from April 2006 to September 2015 were identified using an institutional dataset prepared for the National Cardiovascular Data Registry. Patients were eligible for inclusion if they had symptomatic heart failure with LV ejection fraction ≤35%, an adequate baseline echocardiogram within 365 days and an available electrocardiogram (ECG) within 180 days before CRT implantation, QRS duration ≥120 milliseconds, and LBBB morphology. Patients were excluded if they had a prior CRT device, failed LV lead implantation, 2nd or 3rd degree atrioventricular block at baseline, or cardiac surgery or percutaneous coronary intervention between the baseline echocardiogram and CRT implantation. A subset of patients with a follow-up echocardiogram (ordered for clinical reasons) performed within 60–365 days after CRT implantation were included in secondary analyses.

### Clinical data

Clinical data were obtained from the institutional dataset and through chart review. Patients were categorized as having a history of atrial fibrillation if they had a previous diagnosis of atrial fibrillation or they had atrial fibrillation on the baseline 12-lead ECG. Two authors (D.F. and K.E.), who were blinded to outcomes, reviewed all baseline ECGs and designated QRS morphologies. LBBB morphology was further classified as either strict or non-strict LBBB. [9]

### Echocardiographic analyses

The most recent echocardiogram prior to CRT implantation was analyzed. Measurements of LV end-diastolic and -systolic volumes and two-dimensional speckle-tracked longitudinal strain analyses were performed in the vendor-independent software system ImageArena version 4.6 (TomTec Imaging Systems, Unterschleissheim, Germany) using apical 2-, 3-, and 4-chamber views stored in DICOM format. LV volumes were calculated using a modified Simpson’s triplane method included in the software. The apical 4-chamber view and at least one of the two other views were required for analysis.

For the analysis of longitudinal strain contraction pattern, QRS onset was set as the starting point when possible; when QRS onset was not available in the beginning of the loop, the earliest point available in the cardiac cycle was used. Endocardial borders were traced manually at end-systole, and tracking was assessed visually. If the tracking was considered inadequate, retracing was performed until tracking was deemed correct. The software itself did not provide any information about its assessment of tracking quality.

**Fig. 1:**
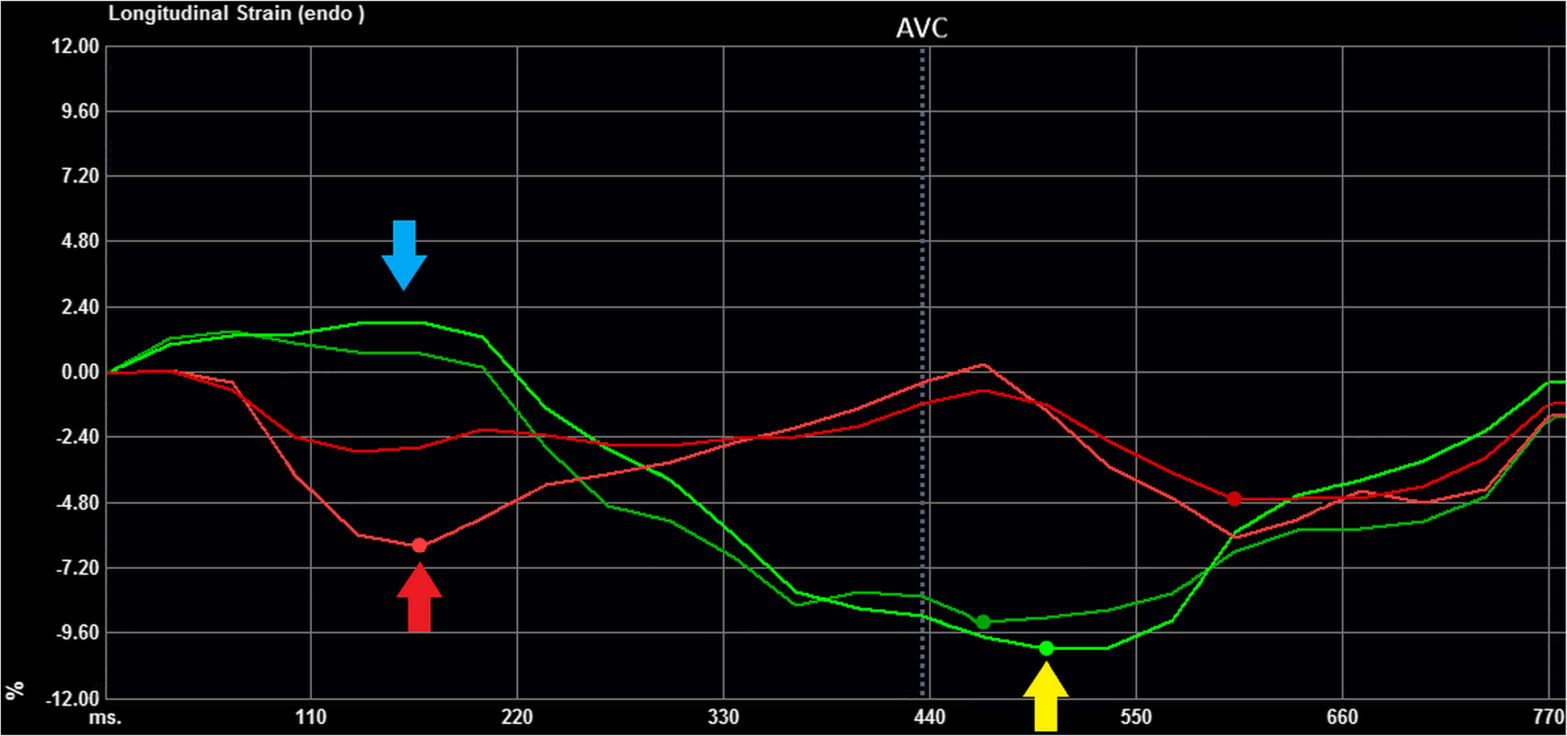
Example of Classical LBBB contraction pattern. The features of a Classical LBBB contraction pattern are the following: 1) Peak shortening of the mid- and/or basal septum (light and dark red lines) within the initial 70% of the ejection phase (red arrow), 2) Initial stretch (blue arrow) of the mid- and/or basal lateral wall (light and dark green lines), and 3) late peak shortening after aortic valve closure (AVC - dotted line) of the mid- and/or basal lateral wall (yellow arrow). The apical segments are usually disregarded, when assessing the Classical LBBB contraction pattern, and therefore they have been omitted from this figure. The dots on each line mark the peak shortening of each segment

Longitudinal strain contraction patterns were classified as a “Classical LBBB contraction pattern” when the septal peak shortening occurred within the initial 70% of the ejection phase, and the lateral wall was initially stretched and had peak shortening after aortic valve closure (Fig. 1) as described by *Risum* et al. [5] Time from QRS onset to aortic valve opening and closure were measured on continuous or pulsed wave spectral Doppler images and manually set accordingly in the strain analysis. All longitudinal strain contraction patterns were read independently by two readers (P.S. and K.E.) blinded to outcome and clinical characteristics. In case of disagreement, the two readers studied the strain images in unison and classified the contraction pattern by consensus. This was done blinded to outcome, clinical characteristics and the initial classifications of each of the readers. The initial reads were used for assessment of inter-reader agreement. A small subgroup of patients had echocardiograms available for analysis of longitudinal strain contraction pattern using vendor-specific software (EchoPAC version 112, GE Healthcare, Chicago, IL, USA), and these were used for assessing agreement between vendor-independent and vendor-specific software.

### Outcomes and analyses

The primary end point was time from CRT implantation to the first event of either death of all causes, heart transplantation or LV assist device implantation. End points were assessed on May 24, 2017 through a query of Duke Enterprise Data Unified Content Explorer (DEDUCE) by incorporating data from hospital billing claims, hospital records, and the United States Social Security Death Index. [10]

Secondary analyses on the subgroup of patients with an eligible follow-up echocardiogram included echocardiographic response defined as a reduction in LV end-systolic volume ≥ 15%, along with relative changes in LV end-diastolic and end-systolic volumes and absolute changes in LV ejection fraction and global longitudinal strain from the baseline to the follow-up echocardiogram.

### Statistical analyses

Normally distributed continuous variables are presented as mean ± standard deviation and differences were tested using the Student *t* test. Non-normally distributed continuous variables are presented as median (25th–75th percentile) and differences were tested using the Wilcoxon rank-sum test. Categorical variables are presented as n (%) and differences were tested using Fisher’s exact test.

Survival free from heart transplantation or LV assist device implantation are presented using Kaplan-Meier curves and differences were tested using the log-rank test. Cox proportional hazards regression was used to estimate hazard ratios in uni- and multivariable analysis of the primary end point. The primary multivariable model included the prespecified covariates QRS duration < 150 milliseconds and ischemic heart disease in accordance with previous literature. [6] A secondary, expanded multivariable model including age, gender, ischemic heart disease, QRS duration < 150 milliseconds, history of atrial fibrillation/flutter, New York Heart Association functional class, creatinine > 1.2 mg/dL, end-systolic global longitudinal strain and use of angiotensin-converting enzyme inhibitor or angiotensin II receptor blocker was also performed to adjust for further potential confounding. Proportional hazards assumptions were checked visually by plotting Schoenfeld’s residuals against time since CRT implantation. No significant violations of the proportional hazards assumptions were observed.

Sensitivity and specificity of a Classical LBBB contraction pattern for echocardiographic response were calculated for the secondary analyses. Intra-reader agreement on longitudinal strain contraction pattern was assessed for one reader (K.E.) by reanalysis of 50 randomly selected patients at least 90 days after the initial read. Inter-reader agreement was assessed using all included patients. For intra- and inter-reader variability and agreement between vendor-independent and vendor-specific software, overall agreement and Cohen’s κ was calculated.

Sensitivity analyses excluding patients without sinus rhythm on the baseline echocardiogram and excluding patients < 25 frames per cardiac cycle were performed.

All statistical analyses were performed in RStudio version 1.1.453 (RStudio, Inc., Boston, MA, USA) running R version 3.5.0 (R Foundation for Statistical Computing, Vienna, Austria). The R package “survival” was used for Cox proportional hazards models and to create Kaplan-Meier curves. [11] A two-sided *p*-value < 0.05 was considered statistically significant.

## Results

**Fig. 2:**
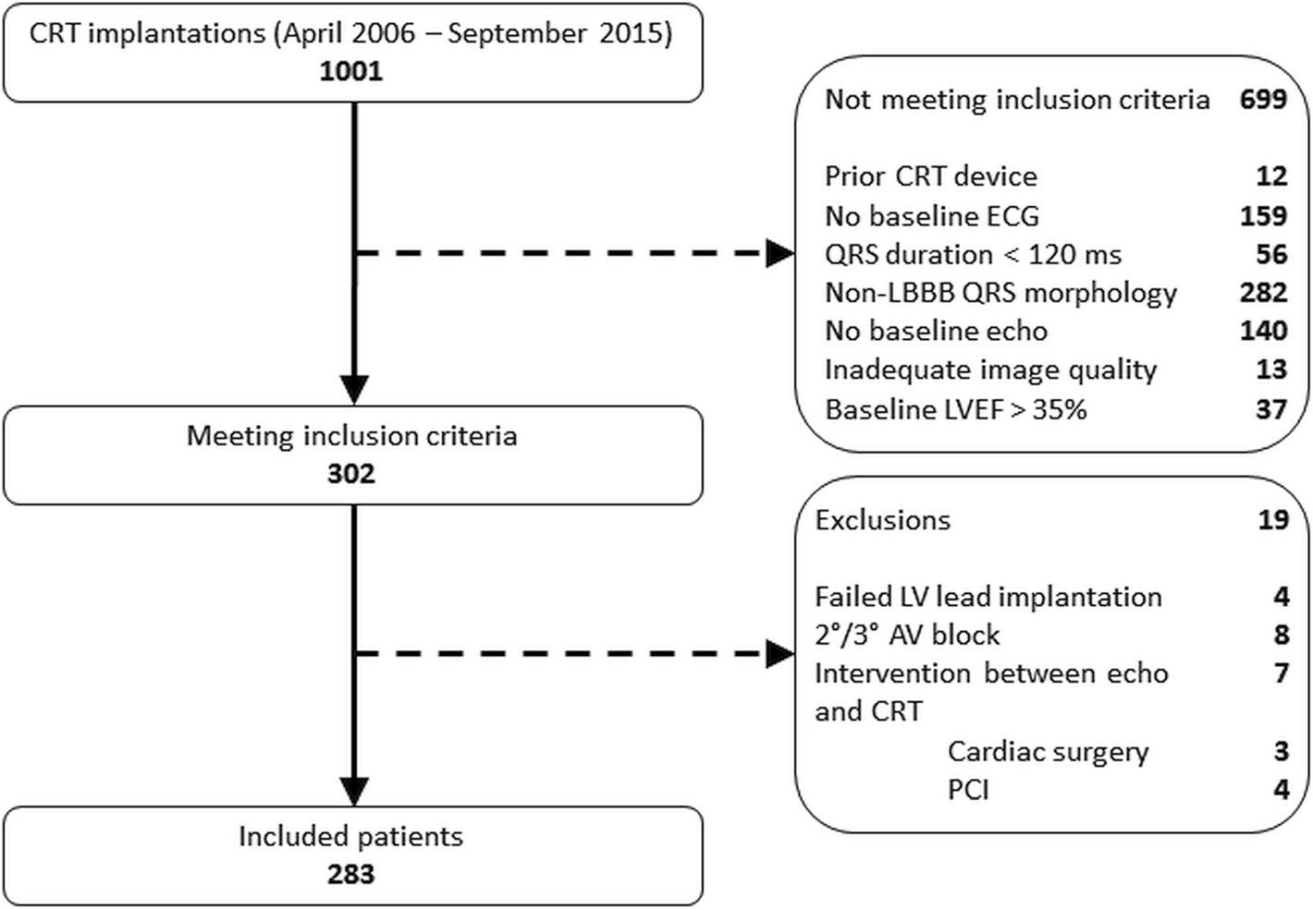
Flowchart of patient inclusion and exclusion process. *AV* Atrioventricular; *CRT* Cardiac resynchronization therapy, *ECG* Electrocardiogram, *echo* echocardiogram; *LBBB* Left bundle branch block, *LV* Left ventricular, *LVEF* Left ventricular ejection fraction; *PCI* Percutaneous coronary intervention

A total of 1001 patients received a CRT device at Duke University Hospital during the period April 2006–September 2015, and 302 of the 1001 met the inclusion criteria. Of these, 283 did not meet any exclusion criteria and were thus included in the study cohort (Fig. 2**)**. The mean age of included patients was 66 ± 11 years, 113 (40%) were women and 136 (48%) had ischemic heart disease. All but 10 (4%) patients had all three apical views available; 9 (3%) were missing the apical 3-chamber view, while 1 (< 1%) was missing the apical 2-chamber view. The median frame rate on analyzed images was 30 frames/second (30–46 frames/second) and the median number of frames per cardiac cycle was 27 (24–37).

### Classical LBBB contraction pattern

Of the 283 included patients, 196 (69%) were classified as having a Classical LBBB contraction pattern. Patients without a classical LBBB contraction pattern were more likely to be male, have ischemic heart disease, have diabetes mellitus, had shorter QRS duration and higher creatinine level (Table 1). Patients with a Classical LBBB contraction pattern had larger LV end-systolic volume, lower LV ejection fraction, and lower end-systolic global longitudinal strain values (Table 2).

**Table 1:** Baseline characteristics

	All	No Classical	Classical	
	*n* = 283	*n* = 87	*n* = 196	*p*-value
Age, years	66 ± 11	67 ± 9	65 ± 12	0.13
Women	113 (40%)	20 (23%)	93 (47%)	< 0.001
Days from ECG to CRT^a^	6 (2–25)	7 (3–24)	6 (1–24)	0.89
Ischemic heart disease	136 (48%)	55 (63%)	81 (41%)	< 0.001
NYHA class III/IV	230 (81%)	74 (85%)	156 (80%)	0.32
QRS duration, ms	157 ± 21	146 ± 17	162 ± 21	< 0.001
QRS duration < 150 ms	110 (39%)	53 (61%)	57 (29%)	< 0.001
Strict LBBB	226 (80%)	55 (63%)	171 (87%)	< 0.001
1st degree AV block	51 (18%)	16 (18%)	35 (18%)	0.99
Atrial fibrillation/flutter	82 (29%)	30 (34%)	52 (27%)	0.20
Hypertension	195 (69%)	67 (77%)	128 (65%)	0.05
Diabetes	90 (32%)	40 (46%)	50 (26%)	< 0.001
Creatinine, mg/dL+	1.2 (1.0–1.5)	1.3 (1.0–1.6)	1.1 (0.9–1.5)	0.01
ACE/ARB	230 (81%)	68 (78%)	162 (83%)	0.32
Betablocker	257 (91%)	79 (91%)	178 (91%)	0.82

^a^Median (25th–75th percentile), Wilcoxon rank-sum test used for testing differences

*ACE* Angiotensin converting enzyme inhibitor, *ARB* Angiotensin II-receptor blocker, *AV* Atrioventricular, *CRT* Cardiac resynchronization therapy, *ECG* Electrocardiogram; *LBBB* Left bundle branch block, *NYHA* New York Heart Association

**Table 2:** Echocardiographic characteristics at baseline

	All	No Classical	Classical	
	*n* = 283	*n* = 87	*n* = 196	*p*-value
Days from echo to CRT^a^	30 (5–78)	22 (5–78)	34 (5–75)	0.89
LVEDV, mL	226 ± 83	214 ± 82	231 ± 83	0.11
LVESV, mL	180 ± 75	164 ± 67	187 ± 78	0.02
LVEF, %	21 ± 7	23 ± 7	20 ± 7	< 0.001
End-systolic GLS, %	−7.0 ± 2.9	−7.9 ± 2.6	−6.7 ± 3.0	0.001
Frame rate ≤ 30 frames/s	159 (56%)	48 (55%)	111 (57%)	0.90
< 25 frames per cardiac cycle	88 (31%)	18 (21%)	70 (36%)	0.01
Sinus rhythm on baseline echo	242 (86%)	75 (86%)	167 (85%)	0.99
Heart rate on baseline echo, min^−1^	77 ± 16	74 ± 14	79 ± 17	0.02

^a^Median (25th–75th percentile), Wilcoxon rank-sum test used for testing differences

*Echo* Echocardiogram, *GLS* Global longitudinal strain, *LVEDV* Left ventricular end-diastolic volume, *LVEF* Left ventricular ejection fraction, *LVESV* Left ventricular end-systolic volume

### Primary outcome

**Fig. 3:**
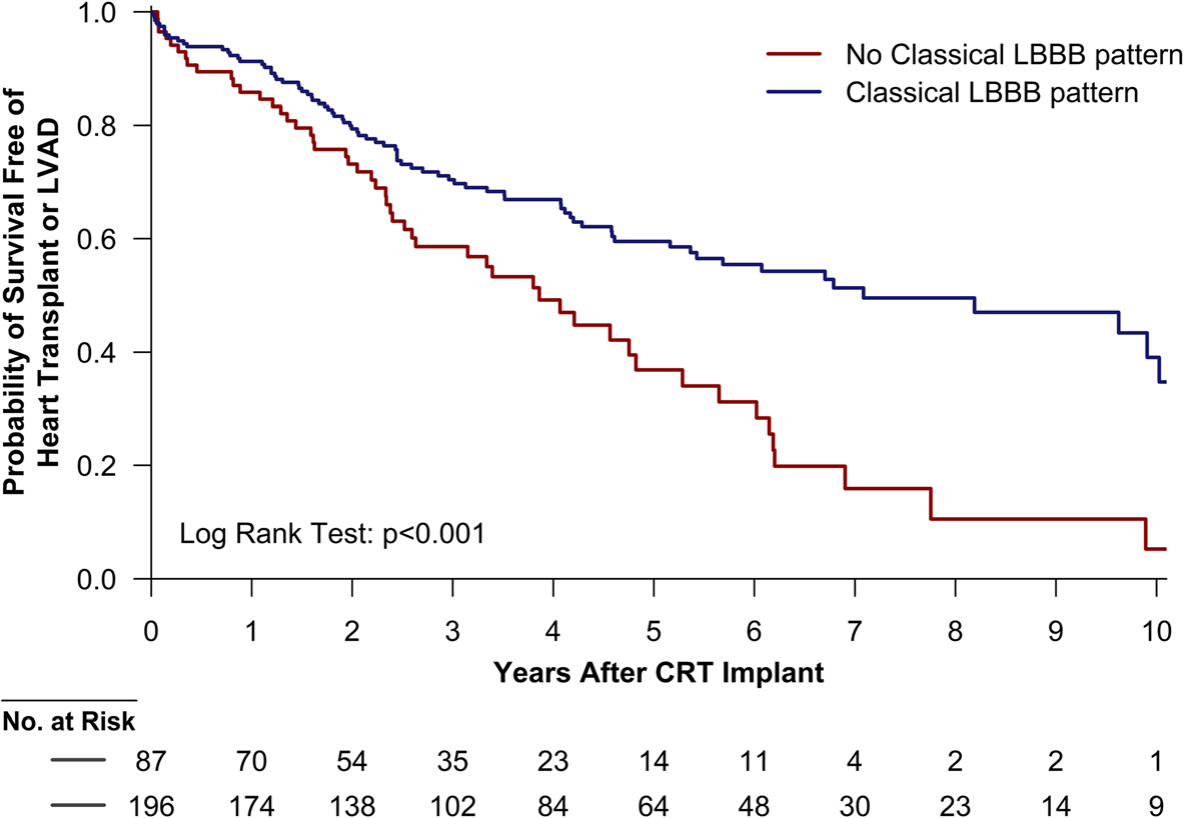
Kaplan-Meier plots for patients with and without a Classical LBBB contraction pattern. Survival free of heart transplantation or LVAD implantation for patients with and without a Classical LBBB contraction pattern CRT = cardiac resynchronization therapy; LBBB = left bundle branch block; LVAD = left ventricular assist device.

A total of 131 (46%) patients reached the composite end point over a median follow-up of 2.8 years (1.8–5.3 years), with death being the most common cause for reaching the end point (*n* = 106, 37%). In patients without a Classical LBBB contraction pattern, 51 of 87 (59%) reached the end point compared to 80 of 196 (41%) of patients with a Classical LBBB contraction pattern (Fig. 3). In univariable Cox regression, the hazard ratio was 1.93 (95% confidence interval, 1.36–2.76, *p* < 0.001) for patients without compared to patients with a Classical LBBB contraction pattern. Adjusted for ischemic heart disease and QRS duration < 150 milliseconds, the hazard ratio was 1.65 (95% confidence interval, 1.12–2.43, *p* = 0.01). Additional adjustment for age, gender, history of atrial fibrillation, New York Heart Association functional class, creatinine > 1.2 mg/dL, end-systolic global longitudinal strain and angiotensin-converting enzyme inhibitor or angiotensin II receptor blocker use yielded a hazard ratio of 1.54 (95% confidence interval, 1.01–2.35, *p* = 0.046).

Sensitivity analysis excluding patients without sinus rhythm on the baseline echocardiogram (*n* = 41, 14%) yielded an adjusted hazard ratio of 1.80 (95% confidence interval, 1.18–2.77). Excluding patients with < 25 frames per cardiac cycle (*n* = 88, 31%) resulted in an adjusted hazard ratio of 1.87 (95% confidence interval, 1.16–3.01). Excluding both patients without sinus rhythm and with < 25 frames per cardiac cycle on the baseline echocardiogram yielded an adjusted hazard ratio of 1.91 (95% confidence interval, 1.14–3.19).

### Echocardiographic outcomes

A follow-up echocardiogram between 60 and 365 days after CRT implantation was available for 123 (43%) of the patients. Median time from CRT implantation to the follow up echocardiogram was 182 days (122–250 days) in patients with a Classical LBBB contraction pattern and 164 days (101–245 days) in patients without (*p* = 0.55). Relative reductions in LV end-diastolic and -systolic volumes, and improvements in LV ejection fraction and end-systolic global longitudinal strain were larger in patients with than in patients without a Classical LBBB contraction pattern (Table 3). Of the 123 patients with a follow-up echocardiogram, 77 (63%) had a relative decrease in LV end-systolic volume ≥ 15% and thus were classified as echocardiographic responders, and 64 of 85 (75%) of patients with compared to 13 of 38 (34%) of patients without a Classical LBBB contraction pattern were echocardiographic responders (*p* < 0.001). Sensitivity and specificity of a Classical LBBB contraction pattern to predict echocardiographic response were 83 and 54%, respectively.

**Table 3:** Changes in LV volumes, LV ejection fraction and end-systolic global longitudinal strain

	No Classical	Classical	
	*n* = 38	*n* = 85	p-value
Relative ΔLV end-systolic volume, %	−7 ± 27	−26 ± 25	< 0.001
vLV end-systolic volume ≤ − 15%	13 (34%)	64 (75%)	< 0.001
Relative ΔLV end-diastolic volume, %	0 ± 24	−16 ± 22	< 0.001
Absolute ΔLV ejection fraction, %-points	7 ± 11	11 ± 10	0.03
Absolute ΔLV end-systolic GLS, %-points	−1.8 ± 4.1	−3.6 ± 3.8	0.02

The changes are from the baseline to the follow-up echocardiogram

*GLS* Global longitudinal strain, *LV* Left ventricular

Sensitivity analyses excluding patients without sinus rhythm or < 25 frames per cardiac cycle on the baseline echocardiogram yielded similar results (data not shown).

### Intra- and inter-reader agreement and agreement between vendor-independent and vendor-specific software

In the randomly selected 50 patients, the intra-reader agreement was 94%, with a Cohen’s κ of 0.86 (95% confidence interval, 0.71–1.00). The inter-reader agreement for the entire cohort was 75% with a Cohen’s κ of 0.42 (95% confidence interval, 0.30–0.54). Thirty-three (12%) patients had an echocardiogram available for analysis of longitudinal strain contraction pattern by vendor-specific software. Of these, 24 (73%) had a Classical LBBB contraction pattern both by vendor-independent and vendor-specific strain analysis. Overall agreement between vendor-independent and vendor-specific software was 88%, with a Cohen’s κ of 0.69 (95% confidence interval, 0.41–0.98).

## Discussion

Using vendor-independent software analysis of standard echocardiographic images, it was found that the presence of a Classical LBBB contraction pattern was associated with better long-term outcome and echocardiographic response after CRT. Furthermore, intra-reader agreement was very good, whereas inter-reader agreement was only moderate. [12]

### Differences between vendor-specific and -independent software

In this study, the association between a Classical LBBB contraction pattern and outcome after CRT was not as strong as in prior studies using vendor-specific software. [5, 6] Furthermore, inter-reader agreement for the Classical LBBB contraction pattern was 75% with a Cohen’s κ of 0.42 in this study compared to inter-reader agreements of 96 and 93% with a Cohen’s κ of 0.87 in the studies using vendor-specific software. [5, 6] Finally, in this study, there was to some extent disagreement between vendor-specific and vendor-independent software. There are several possible explanations for this. The differences may be caused by the different methods used by the software algorithms. Another possible explanation is related to the different image formats used by the different software. Vendor-specific software has the possibility of analyzing raw data, whereas vendor-independent software (at least in this study) analyzed images in DICOM format. More than half of these were temporally compressed to 30 frames/second as well as spatially compressed, whereas strain analysis using vendor-specific software is often performed at a frame rate > 60 frames/second. [5, 6, 13]

A recent study found that at least 25–30 frames per cardiac cycle is necessary for accurate measurement of peak global longitudinal strain. [14] It would be reasonable to assume that at least the same number of frames per cardiac cycle are necessary to accurately determine regional strain values and curves. The temporal compression of the DICOM images to 30 frames/second means that 25 frames per cardiac cycle will not be available in patients with a heart rate > 72 /min, resulting in an underestimation of the regional strain values and a blunting of the regional strain curves. Ultimately, this could cause rapid changes in the cardiac contraction to be missed, for instance the early peak contraction of the septum, and thus affect the overall interpretation of the longitudinal strain contraction pattern. It could also lead to difficulties in determining the actual 70% cutoff point for the early septal peak contraction. The performed sensitivity analysis excluding patients with < 25 frames per cardiac cycle yielded higher hazard ratios, which supports that the compressed frame rate explains part of the discrepancy between the current and prior studies.

The spatial compression of DICOM images means that fewer data points are available for the software, decreasing the ability to discriminate and increasing the susceptibility to noise. The combination of blunted strain curves and a higher susceptibility to noise can produce contraction patterns that are difficult to interpret and may explain the lower inter-reader agreement in this study compared to studies using vendor-specific software. [5, 6]

### Activation delay and outcome after cardiac resynchronization therapy

Evidence suggests that a substantial proportion of CRT recipients do not improve their clinical or echocardiographic status. [3, 4] The reasons for lack of improvement after CRT are multiple and complex. However, studies suggest that not all patients thought to have LBBB have a LV activation delay, [15, 16] and this may explain why some CRT recipients do not improve. Therefore, there has been a massive interest in identification of LV activation delay. The Classical LBBB contraction pattern is one such approach. It identifies the mechanical consequences of LBBB with opposing movements of the LV septum and free wall, and studies suggest that patients exhibiting the Classical LBBB contraction pattern benefit the most from CRT. [5, 6] This is further supported by studies finding similar associations between variations of the Classical LBBB contraction pattern with outcome in CRT recipients. [17–20] The results in the current study are in parallel with these studies, albeit the association is not as strong despite comparable sample sizes. This study performed strain analyses retrospectively on echocardiograms obtained for clinical indications and rarely optimized for strain analysis, whereas the other studies used prospectively collected data with echocardiograms optimized for strain analysis. [5, 6, 13, 17] Based on the current results, retrospective application of longitudinal strain contraction pattern using vendor-independent software on echocardiograms stored in compressed DICOM format cannot be recommended for clinical decision making in potential CRT candidates.

### Standardization of speckle-tracked strain

It is recognized that differences between the software used for speckle-tracked strain, is a major limitation of the clinical use of strain analyses, and therefore a task force was made in a cooperation between the European Association of Cardiovascular Imaging, American Society of Echocardiography, and industry representatives with the mission of standardizing speckle-tracked deformation analysis. [21] So far, their work has mainly focused on the quantitative aspects of strain measurements, and there has been less focus on qualitative aspects like contraction patterns. [8, 22, 23] A recent study of CRT recipients found that there were significant differences between vendor-specific and vendor-independent software for both qualitative and quantitative measures of mechanical dyssynchrony. [24] Furthermore, dyssynchrony parameters derived from vendor-independent software had a weaker association with echocardiographic response than parameters derived from vendor-specific software. [24] These results support the notion that qualitative assessment of LBBB contraction pattern may be less accurately determined with vendor-independent software.

### Limitations

This was a retrospective cohort study with no control group of patients that did not receive CRT. Thus, it is not possible to assess the effect of CRT in patients with or without a Classical LBBB contraction pattern. Furthermore, the retrospective design limited the study to include only patients who had a baseline echocardiogram performed at Duke University Hospital. Selection bias in this subgroup of CRT recipients is very probable, however it is unlikely to affect the associations between contraction pattern and outcome after CRT. In addition, echocardiographic follow-up data were only available for those who had an echocardiogram ordered for clinical reasons, and the time from CRT implantation to the follow-up echocardiogram was therefore inconsistent. This means that echocardiographic response rate and magnitudes of volume reductions may not be generalizable. Furthermore, the analyses were performed retrospectively on the available echocardiographic images, which were not always optimized for speckle-tracked strain analysis, and it is not possible to determine if the modest performance of vendor-independent strain software analysis is due to image compression, image acquisition, or the software itself. Finally, a comparison between the performance of vendor-independent and vendor-specific software regarding the Classical LBBB contraction pattern and its association with CRT outcome was not possible, due to the limited number of patients with a baseline echocardiogram allowing for strain analysis using vendor-specific software.

## Conclusion

A classical LBBB contraction pattern derived from two-dimensional speckle-tracked longitudinal strain by vendor-independent software is associated with improved survival free from heart transplantation and LV assist device implantation and with higher probability of echocardiographic response. However, inter-reader agreement was only moderate, which diminishes the clinical utility of vendor-independent software for decision making in potential CRT candidates.

## Abbreviations

CRT: cardiac resynchronization therapy
DICOM: Digital Imaging and Communications in Medicine
ECG: electrocardiogram
LBBB: left bundle branch block
LV: left ventricle/ventricular
